# Long non-coding RNA LOC107985656 represses the proliferation of hepatocellular carcinoma cells through activation of the tumor-suppressive Hippo pathway

**DOI:** 10.1080/21655979.2021.1984005

**Published:** 2021-10-08

**Authors:** Yu Zeng, Qin Xu, Nan Xu

**Affiliations:** a Department of Internal Medicine, Chenglong Campus Hospital, Sichuan Normal University, Sichuan Province China; b Department of Infectious Diseases, First Affiliated Hospital of Xinjiang Medical University, Xinjiang China; c Department of Infectious Diseases, West China Hospital of Sichuan University, Sichuan Province China

**Keywords:** Lncrna, loc107985656, lats1, miR-106b-5p, hippo pathway, hcc

## Abstract

Long non-coding RNAs (lncRNAs) play important regulatory roles in hepatocellular carcinoma (HCC). However, the function of LOC107985656 in HCC progression remains unclear. The lncRNA, mRNA and miRNA levels in HCC tissues or cells were measured using real-time quantitative polymerase chain reaction (RT-qPCR). The proliferation of cancer cells was evaluated using 3-(4, 5-dimethyl-2-thiazolyl)-2,5-diphenyl-2-H-tetrazolium bromide (MTT) viability and colony formation assays. Bioinformatics prediction, dual luciferase assay and RNA pull-down assay were performed to analyze the relationships between LOC107985656 and miR-106b-5p, or miR-106b-5p and large tumor suppressor 1 (LATS1). The protein expression levels were detected using Western blot. Results showed that LncRNA LOC107985656 was downregulated in HCC tissues and cells. Upregulation of LOC107985656 inhibited the proliferation of HCC cells, whereas its knockdown promoted this phenomenon. LOC107985656 could activate the tumor-suppressive Hippo pathway by repressing yes association protein (YAP) and WW domain-containing transcription regulator protein 1 (WWTR1, also known as TAZ) (two homologs of Yki) protein expression in HCC. Further investigation suggested that LOC107985656 regulated the expression of LATS1 by acting as a sponge for absorbing miR-106b-5p in HCC cells. In conclusion, this study unraveled the role of LOC107985656 following a ceRNA (competing endogenous RNAs) mechanism for the miR-106b-5p/LATS1 axis in HCC. The results indicate potential diagnostic and therapeutic applications of LOC107985656 in HCC.

Abbreviations:

HCC: hepatocellular carcinoma; LncRNA: long non-coding RNA; LATS1: large tumor suppressor 1; MTT: 3-(4, 5-dimethyl-2-thiazolyl)-2,5-diphenyl-2-H-tetrazolium bromide; YAP: yes association protein; WWTR1: WW domain-containing transcription regulator protein 1; cDNA: single‐stranded complementary DNA; RT-qPCR: real-time quantitative polymerase chain reaction; Radio-Immunoprecipitation Assay (RIPA); BCA: bicinchoninic acid; ASO: antisense oligonucleotide; MST1/2: Ste20-like kinases 1/2; TEAD: TEA domain transcription factor; ceRNA: competing endogenous RNAs.

## Introduction

Hepatocellular carcinoma (HCC) is a common primary liver cancer with a high mortality rate worldwide [1,2]. The early treatment of HCC has developed rapidly and made significant progress, which has improved the survival rate of patients; however, the overall 5-year survival rate of HCC patients remains low [3,4]. Many patients are diagnosed at the middle or late stage of the disease, which cannot be cured because of tumor recurrence [5,6]. Thus, the molecular mechanisms underlying HCC progression must be elucidated, and potential diagnostic or therapeutic strategies for HCC treatment must be developed.

Long non-coding RNA (lncRNA) molecules are a class of long non-coding RNAs with over 200 nucleotides (nt) that cannot encode proteins [7,8]. An increasing number of studies have shown that lncRNAs play an essential role in regulating physiological or pathological processes. In particular, some lncRNAs are involved in the occurrence and development of various cancers [9–11]. Although several lncRNAs have been demonstrated in HCC, many lncRNAs with unknown functions remain [12,13]. For example, LOC107985656 is a newly reported lncRNA whose role and molecular mechanism in HCC remain unclear.

In the present study, LOC107985656 was downregulated in HCC tissues and cells. In addition, LOC107985656 participated in suppressing the proliferation of HCC cells in vitro. Furthermore, LOC107985656 activated the Hippo pathway via regulating the miR-106b-5p/LATS1 axis. These results suggest that LOC107985656 is a potential biomarker or therapeutic target for HCC.

## Materials and methods

### Tissue samples

Thirty HCC and corresponding normal liver tissues were collected from patients undergoing routine surgery in the West China Hospital of Sichuan University from 2016 to 2020. All cases were histologically confirmed to be HCC, and the patients were unprepared for radiotherapy and/or chemotherapy. These tissue samples were collected and quickly frozen in liquid nitrogen and stored at −80°C. The prior written and informed consent from each of the studies has been approved by the Ethics Committee of West China Hospital of Sichuan University (2020–0567).

### Cell lines and culture

HCC cell lines Huh7, SMMC‐7721, HepG2.2.15 and HepG2 were obtained from the American Type Culture Collection. The immortalized normal human hepatocyte cell line LO2 was obtained from the Cell Bank of Type Culture Collection (Chinese Academy of Sciences, Shanghai, China).

Cell lines Huh7, SMMC‐7721, HepG2.2.15 and HepG2 were cultured in Dulbecco’s modified Eagle’s medium (DMEM) containing 10% fetal bovine serum (FBS) (Gibco, Grand Island, NY, USA). Cell line LO2 was cultured in RPMI 1640 medium containing 10% FBS. The cells were incubated at 37°C with 5% CO_2_. All cell lines were mycoplasma free.

### RNA isolation and RT-qPCR

TRIzol reagent (Thermo Fisher, United States) was used to isolate total RNA from tissues and cells. mRNA was reverse transcribed into single‐stranded complementary DNA (cDNA) by using the PrimeScript RT-PCR kit (TaKaRa, Dalian, China) in accordance with the manufacturer’s instructions. LOC107985656 and LATS1 levels were measured using 2 × SYBR Green PCR Mastermix (Solarbio, Beijing, China) in accordance with the manufacturer’s instructions, and glyceraldehyde-3-phosphate dehydrogenase (GAPDH) served as the internal reference. The level of miR-106b-5p was measured through RT-qPCR using TaqMan™ MicroRNA Assay (CAS#: 4427975, Applied Biosystems, Forster City, USA), and U6 served as an internal reference. The results were quantified using the 2^− ΔΔCt^ method. The primers used in this study were as follows: LOC107985656: 5ʹ-CAGAGGACCCCAGAGGATCA-3ʹ (Forward) and 5ʹ-GTGTCAGGAGAGCCAGCAAT-3ʹ (Reverse); LATS1: 5ʹ- AATTTGGGACGCATCATAAAGCC-3ʹ (Forward) and 5ʹ- TCGTCGAGGATCTTGGTAACTC-3ʹ (Reverse); YAP: 5ʹ- ACCCACAGCTCAGCATCTTC-3ʹ (Forward) and 5ʹ- ATTCCTGAGACATCCCGGGA-3ʹ (Reverse); TAZ: 5ʹ- CACCGTGTCCAATCACCAGTC-3ʹ (Forward) and 5ʹ- TCCAACGCATCAACTTCAGGT-3ʹ (Reverse); GAPDH: 5ʹ-GATTTGGTCGTATTGGGCGC-3ʹ (Forward) and 5ʹ-AGTGATGGCATGGACTGTGG-3ʹ (Reverse); miR-106b-5p: 5ʹ-TGCGG TAAAGTGCTGACAGTGC-3ʹ and 5ʹ-CCAGTGCAGGGTCCGAGGT-3ʹ (reverse); U6: 5ʹ-TGCGGGTGCTCGCTTCGGCAGC-3ʹ (Forwa-rd) and 5ʹ-CCAGTGCAGGGTCCGAGGT-3ʹ (Reverse).

### Cell transfection

Cells were grown in a 6-well plate and were transfected with Lipofectamine 2000 reagent (Invitrogen, California, USA) when the cell amount reached about 70%–80% confluence. Cell transfection was performed in accordance with the manufacturer’s instructions.

### MTT assay

Cell viabilities were detected using the MTT assay. After transfection, the cells were seeded onto a 96-well plate with a density of 3000 cells/well. The transfected cells were detected using the MTT assay after transfection for 48–96 h. Then, 10 μL of MTT solutions were added to each well and continued to culture for 4 h. The supernatant was discarded, and 100 μL of DMSO was added to dissolve for 5 min without light. The absorbance was detected with a microplate reader at 490 nm.

### Colony formation assay

After transfection, the cells were seeded onto a 12-well plate with a density of 300 cells/well. The cells were consecutively grown for 10–14 days at 37°C. Each well was washed using PBS, and the cells were fixed with 4% paraformaldehyde before staining with 0.5% crystal violet. Formed colonies containing more than 50 cells were

counted and recorded for statistical analysis.

### Transwell assay

Cells in each group were digested using trypsin, neutralized by adding serum-containing DMEM and then gently mixed evenly with a pipette and centrifuged at 2000 rpm for 2 min. The supernatant was discarded, and serum-free DMEM was used for re-suspension. The cell concentration was adjusted to 1 × 10^5^ cells/mL. For the invasion experiment, 2 × 10^5^ cells were added into the upper chamber of the Transwell. For the migration experiment, 30 μL of Matrigel (1 mg/mL) was added to the upper chamber of Transwell, followed by 4 × 10^5^ cells. Afterward, 600 μL of 20% serum DMEM was added to the basolateral chamber, cultured for about 48 h, fixed with 4% paraformaldehyde for 20 min and stained with crystal violet for 5 min. Photographs were taken under a microscope, and the number of cells per field was counted.

### Western blot analysis

After transfection, the protein was extracted from the cells by using Radio-Immunoprecipitation Assay (RIPA) Lysis Buffer (Solarbio, China) containing protease inhibitor Cocktail (Biosharp, China). The concentrations of extracted proteins were detected with a bicinchoninic acid (BCA) protein assay kit (Biosharp, China). First, 30 μg of proteins were separated by 10% SDS PAGE and then transferred onto 0.45 μm PVDF membranes (Biosharp, China). The PVDF membranes were blocked in 5% skim milk, and an appropriate concentration of primary antibodies was added and incubated overnight at 4°C. The primary antibodies used were as follows: YAP (CST, 1:1000, CAS:14074), TAZ (CST, 1:1000, CAS: 72,804), p-YAP (CST, 1:1000, CAS: 13,008), p-TAZ (CST, 1:500, CAS: 59,971), LATS1 (CST, 1:1000, CAS: 9153), GAPDH (Proteintech, 1:5000, CAS: 60,004-1-Ig). After washing three times with TBST, the HRP secondary antibody was used to incubate with the membranes at room temperature for 1 h. Finally, the signals were probed with ECL solution exposure and quantified by ImageJ software. GAPDH was used as an internal reference.

### Dual-luciferase reporter assay

HCC cells were seeded onto 24-well plates for 24 h. Then, the cells were co-transfected with pGL3 luciferase vector carrying wild-type (WT) or mutated (Mut) sequences and miR-106b-5p mimics or antisense oligonucleotide of miR-106b-5p (ASO-miR-106b-5p) by using Lipofectamine® 2000 (Invitrogen; Thermo Fisher Scientific, Inc.). Luciferase activities were measured using a dual-Luciferase Reporter Assay system (Promega, Madison, WI, USA) at 48 h after transfection in accordance with the manufacturer’s instructions. The relative luciferase activity was quantified by using Renilla luciferase activity as an internal reference.

### RNA pull-down assay

RNA pull-down assay was performed according to the method previously reported with minor modifications [14]. Briefly, for the miR-106b-5p pull-down assay, first, 3ʹ biotin-labeled miR-106b-5p (miR-106b-5p) and control probes were synthesized and labeled by General Biological Systems (Anhui, China) Co., LTD. Then, these probes were transfected into Huh7 cells. After 48 h from transfection, whole cells were harvested. Then, RIPA Lysis Buffer (Beyotime, China), protease inhibitor and RNase inhibitor were added to the cells. The total RNA was pretreated with DNase I by heating at 65°C for 5 min and then given an ice bath before its incubation with streptavidin-coated magnetic beads (Beyotime, China) at 4°C for 4 h. Finally, the microbeads were washed twice using RIPA Lysis Buffer, and the total RNA was isolated using an RNA Extraction Kit (TaKaRa, China). RT-qPCR was used to detect the mRNA levels of LOC107985656 and LATS1.

### Statistical analysis

All experiments were performed at least three times independently, and data are presented as the mean ± SD. Statistical analyses were performed using GraphPad Prism 7 (GraphPad Software, Inc.). Unpaired Student’s t-test was used to compare the significant difference between the two groups, and one-way ANOVA followed by Tukey’s post hoc test was used to compare the data among multiple groups. Statistical significance was considered at P < 0.05.

## Results

### LOC107985656 was downregulated in patients with HCC and HCC cell lines

**Figure 1.**
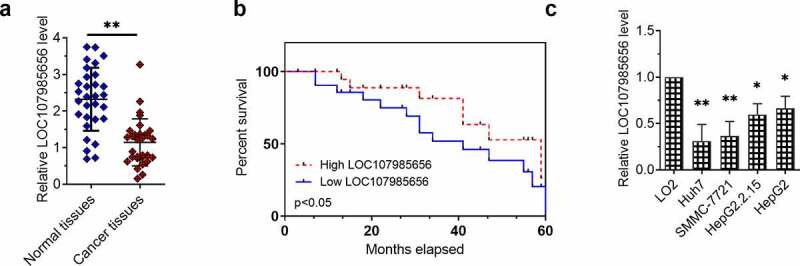
**LOC107985656 was downregulated in HCC tissues and cells**. (a) Relative expression of LOC107985656 in HCC tissues (n = 30) and corresponding non-tumor tissues (n = 30) according to RT-qPCR results. (b) Kaplan–Meier survival analysis demonstrating that the low expression of LOC107985656 was associated with poor prognosis in patients with HCC. (c) RT‐qPCR results showing that LOC107985656 was downregulated in HCC cell lines (Huh7, SMMC‐7721, HepG2.2.15, and HepG2) compared with immortalized normal human hepatocyte cell line LO2. *p < 0.05, and **p < 0.01

The expression of LOC107985656 in the HCC tissues (n = 30) and paired non-tumor tissues (n = 30) was first determined by RT-qPCR to explore its role in HCC. The expression of LOC107985656 was significantly lower in the cancer tissues compared with the paired non-tumor tissues (Figure 1(a)). Kaplan–Meier survival analysis results suggested that a low expression of LOC107985656 foreboded the poor prognosis of patients with HCC (Figure 1(b)). Furthermore, the expression of LOC107985656 was lower in Huh7, SMMC‐7721, HepG2.2.15 and HepG2 cell lines compared with immortalized normal human hepatocyte cell line LO2. RT-qPCR results also showed that the expression of LOC107985656 was markedly lower in HCC cells (Huh7, SMMC‐7721, HepG2.2.15 and HepG2) than in immortalized normal human hepatocyte cell line LO2 (Figure 1(c)). The data suggest that the downregulation of LOC107985656 in patients with HCC indicates a poor prognosis.

### LOC107985656 repressed cell proliferation and metastasis of HCC cells

**Figure 2.**
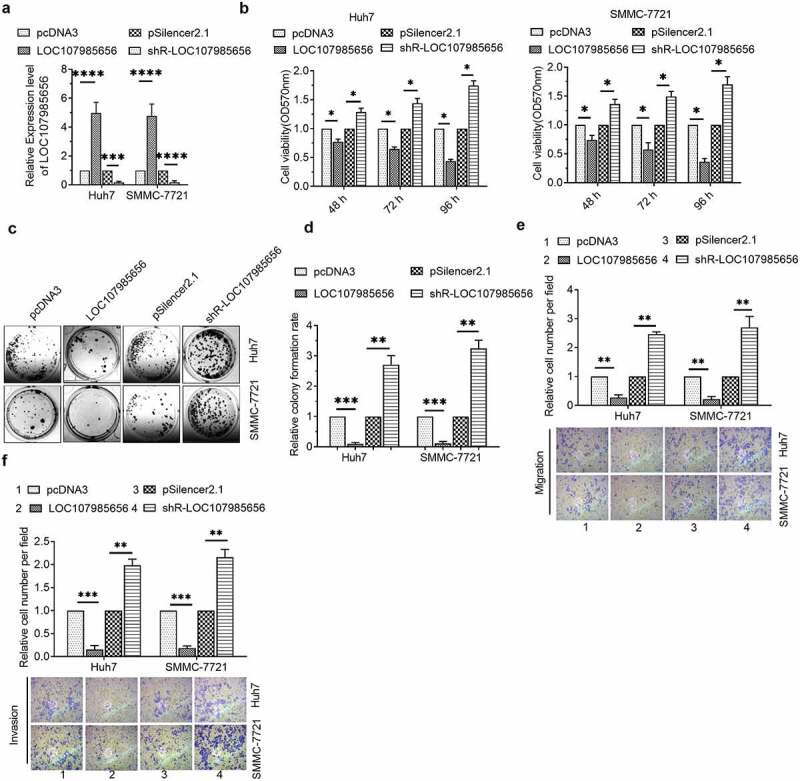
**LOC107985656 inhibited the proliferation of HCC cells**. (a) RT‐qPCR of the expression of LOC107985656 in HCC cells after transfection with indicated plasmids. (b) MTT assay of the viabilities of HCC cells induced by LOC107985656 overexpression or knockdown. (c and d) Effects of LOC107985656 overexpression and knockdown on the proliferation of HCC cells as analyzed by colony formation assay. *p < 0.05, **p < 0.01, ***p < 0.001, and ****p < 0 .0001

We next showed whether or not LOC107985656 could regulate the viabilities of HCC cells. RT‐qPCR was first used to confirm the efficacy of LOC107985656-overexpressing or -knockdown plasmids (Figure 2(a)). MTT assay results revealed a decrease in the viabilities of HCC cells transfected with the LOC107985656-overexpressing plasmid and an increase in the viabilities of HCC cells transfected with shR-LOC107985656 (Figure 2(b)). In addition, colony formation results showed that upregulation of LOC107985656 inhibited the colony formation of HCC cells, whereas an opposite result was observed by knockdown of LOC107985656 (Figure 2(c,d)). We further investigated whether this lncRNA regulated cancer cell migration or invasion in HCC. Transwell migration and invasion experiments results showed that overexpression of LOC107985656 repressed the abilities of cell migration and invasion, whereas knockdown of LOC107985656 showed an opposite result in HCC cells (Figure 2(e,f). These results suggest that LOC107985656 represses the proliferation and metastasis of HCC cells.

### LOC107985656 regulated the tumor-suppressive Hippo pathway

**Figure 3.**
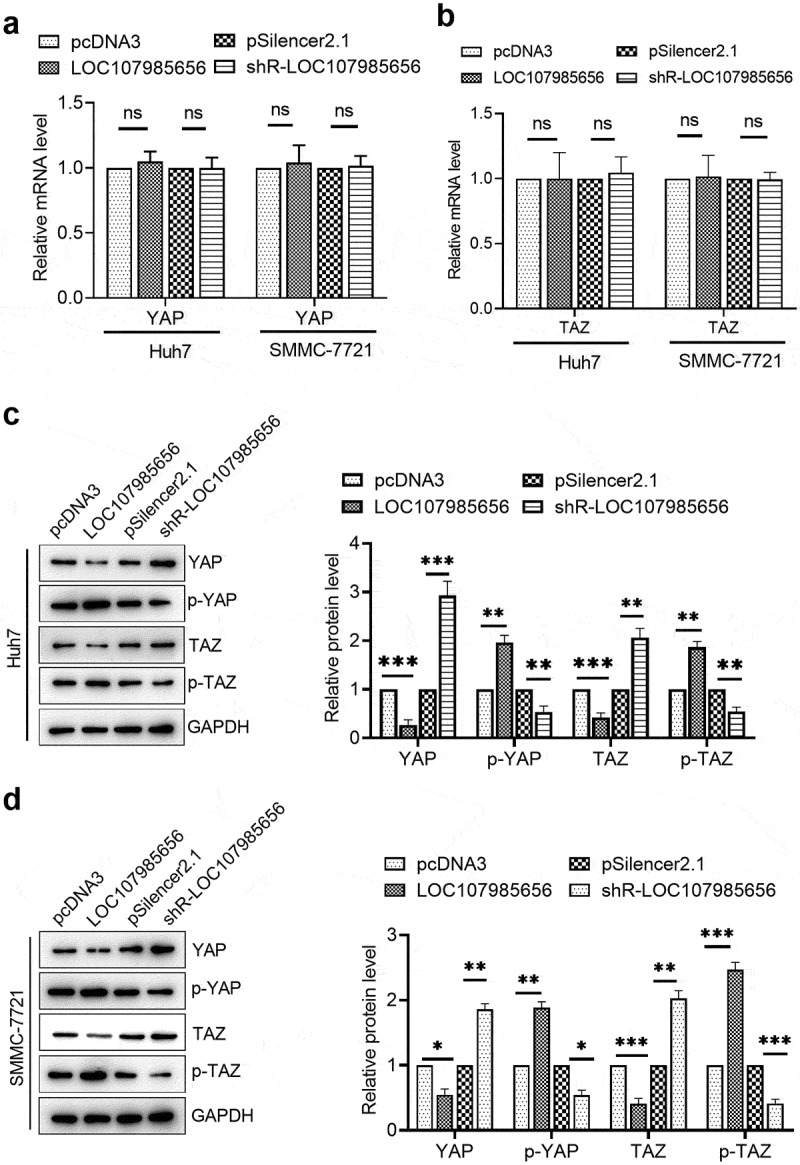
**LOC107985656 activated the tumor-suppressive Hippo pathway**. (a) RT‐qPCR of the mRNA level of YAP and TAZ in HCC cells after transfection with LOC107985656 overexpression and knockdown plasmids. (b) Western blot of YAP, p-YAP, TAZ and p-TAZ expression in HCC cells. *p < 0.05, **p < 0.01, ***p < 0.001; ns, no significance

Accumulated evidence shows that Hippo signaling is a key regulator, and activation of the Hippo pathway acts as a tumor-suppressive in several human cancers, for example, gastric cancer [15], lung cancer [16], and HCC [17–20], etc. YAP and TAZ, the effectors of the Hippo pathway, are transcription co-activators that serve as oncogenes in tumorigenesis and development in several cancers [21]. We evaluated the mRNA and protein levels of YAP and TAZ in HCC cells to assure whether or not LOC107985656 could regulate the Hippo pathway. RT-qPCR results showed that overexpression or silencing of LOC107985656 could not affect the transcriptional levels of YAP and TAZ (Figure 3(a,b)). However, the protein levels of YAP and TAZ were significantly reduced by LOC107985656 overexpression. Conversely, an increase in YAP and TAZ protein expression was found after LOC107985656 knockdown (Figure 3(c,d)). Furthermore, we observed that phosphorylated YAP and TAZ increased when LOC107985656 was overexpressed; on the contrary, LOC107985656 knockdown significantly repressed the levels of phosphorylated YAP and TAZ (Figure 3(c,d)). These results suggest that LOC107985656 could activate the tumor-suppressive Hippo pathway through increasing the phosphorylation of YAP/TAZ in Hippo pathway and reducing total YAP/TAZ protein levels.

### LOC107985656 promoted the expression of LATS1

**Figure 4.**
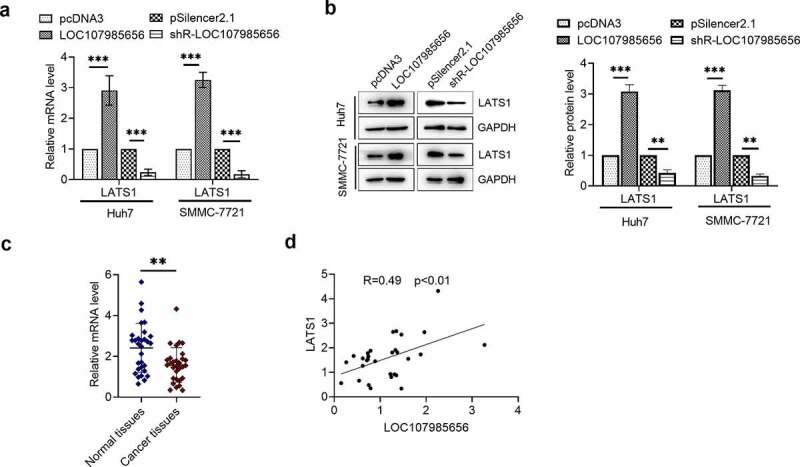
**LOC107985656 upregulated the expression of LATS1**. (a) RT‐qPCR of the mRNA level of LATS1 in HCC cells after transfection with LOC107985656 overexpression and knockdown plasmids. (b) Western blot assay results of the protein level of LATS1 in HCC cells. (c) RT-qPCR of the relative mRNA level of LATS1 in HCC tissues (n = 30) and corresponding non-tumor tissues (n = 30). (d) Positive correlation between LOC107985656 mRNA levels and LATS1 mRNA levels in HCC tissues. **p < 0.01, ***p < 0.001, and ****p < 0 .0001

To further investigate the mechanism of LOC107985656 in HCC, we confirmed whether or not LATS1, the major kinase component of the Hippo pathway, plays a pivotal role in the LOC107985656-mediated regulation of the Hippo pathway in HCC cells. RT-qPCR results showed that LATS1 mRNA level increased in the HCC cells upon the overexpression of LOC107985656 and significantly reduced upon the knockdown of LOC107985656 (Figure 4(a)). Western blot results revealed a significant increase in LATS1 protein expression in the cells transfected with the LOC107985656 overexpressing plasmid and a reduction in LATS1 protein expression in the cells transfected with shR-LOC107985656 (Figure 4(b)). Furthermore, RT-qPCR results showed that the LATS1 mRNA level was lower in the HCC tissues (n = 30) than in the paired non-tumor tissues (n = 30) (Figure 4(c)). Notably, we observed a positive correction between the mRNA levels of LOC107985656 and LATS1 in the HCC tissues (Figure 4(d)). These results suggest that LOC107985656 activates the Hippo pathway by regulating the expression of LATS1 in HCC.

### LOC107985656 acted as a ceRNA to regulate the expression of LATS1

**Figure 5.**
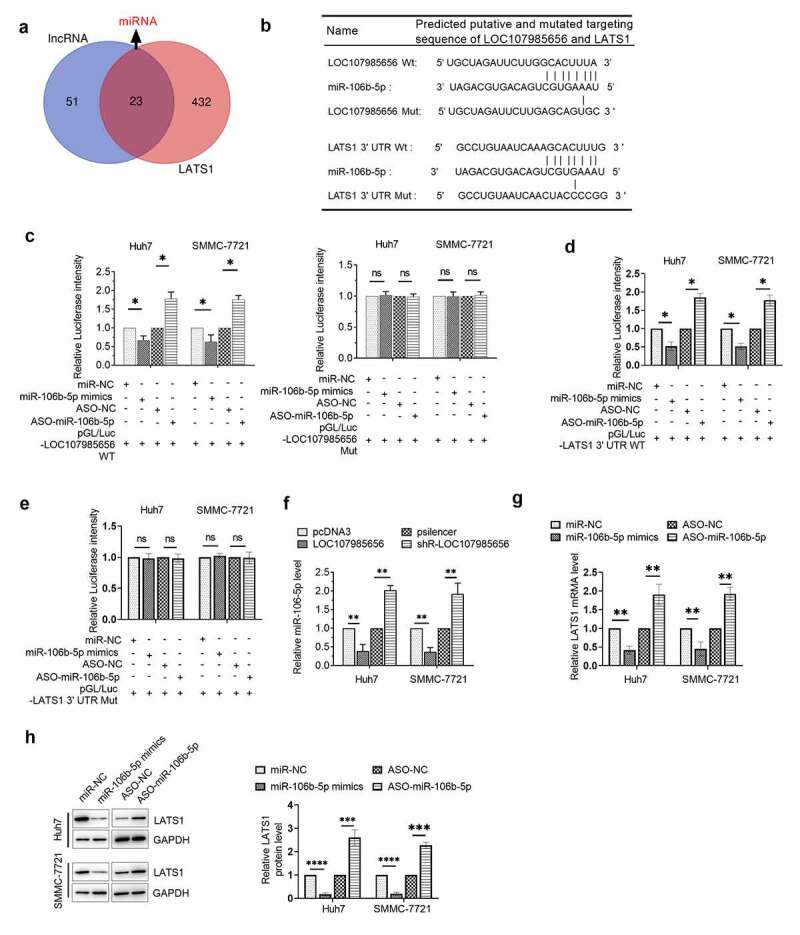
**LOC107985656 upregulated the expression of LATS1 by sponging miR-106b-5p**. (a) Venn diagram of potential miRNAs that targeted LOC107985656 and LATS1 (overlapping fraction). miRNAs that targeted LOC107985656 and LATS1 were predicted by an online database miRDB (http://mirdb.org/) and TargetScanHuman 7.2 (http://www.targetscan.org/vert_72/), respectively. (b) Predicted binding sites for miR-106b-5p in LOC107985656 and the 3ʹUTR of LATS1 as well as the mutations in the binding sites. (c) Luciferase activity in HCC cells co-transfected with pGL/Luc- LOC107985656 wild type or pGL/Luc-LOC107985656 mut with miR-106b-5p mimics or ASO-miR-106b-5p. (d) Luciferase activity in HCC cells co-transfected with pGL/Luc-LATS1 3ʹUTR wild type or pGL/Luc-LATS1 3ʹUTR mut with miR-106b-5p mimics or ASO-miR-106b-5p. (g and h) RT‐qPCR and Western blot assay of the mRNA and protein levels of LATS1 in HCC cells after transfection with miR-106b-5p mimics or its inhibitors ASO-miR-106b-5p. *p < 0.05, **p < 0.01, ***p < 0.001, and ****p < 0 .0001; ns, no significance

Although we have demonstrated that LOC107985656 could regulate the expression of LATS1, the underlying mechanism remains unclear. Thus, we investigated whether or not LOC107985656 could increase the expression of LATS1 through a ceRNA mechanism. MiRNAs that might bind to LOC107985656 and the 3ʹUTR of LATS1 were predicted by an online database miRDB and TargetScanHuman 7.2, respectively. Venn diagram results suggested that 23 miRNAs target both LOC107985656 and LATS1 (Figure 5(a)). Among these miRNAs, miR-106b-5p, which has predicted binding sites in LOC107985656 and the 3ʹUTR of LATS1 (Figure 5(b)), was selected for further investigation because of its possible oncogenic role in HCC via regulating several target genes, such RUNX3 [22], PTEN [23] and FOG2 [24]. The results of the dual-luciferase reporter assay showed that miR-106b-5p could target LOC107985656 and the 3ʹUTR of LATS1 in HCC cells (Figure 5(c,e). Furthermore, RNA pull-down assay was used to demonstrate the interaction between LOC107985656 and miR-106b-5p, as well as the interaction between miR-106b-5p and LATS1 (Figure (s1)). In addition, overexpression of LOC107985656 markedly reduced the expression of miR-106b-5p in HCC cells, whereas silencing of LOC107985656 significantly increased its expression (Figure 5(f)). Finally, increasing miR-106b-5p levels could significantly reduce the mRNA and protein levels of LATS1 in HCC cells, whereas miR-106b-5p inhibitor could markedly increase these levels (Figure 5(g,h)). The results imply that LOC107985656 regulates the expression of LATS1 by acting as a sponge for absorbing miR-106b-5p in HCC cells.

### LOC107985656 regulated the miR-106b-5p/LATS1 axis to activate the Hippo pathway

**Figure 6.**
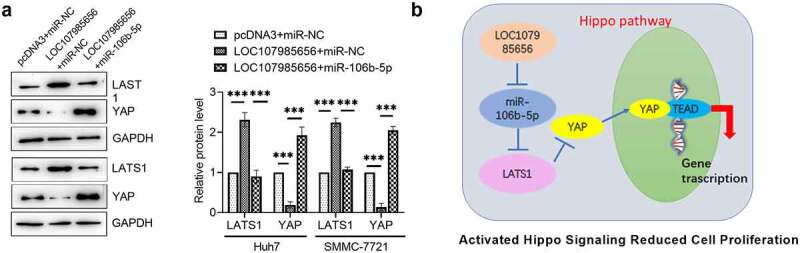
**LOC107985656 activated the Hippo pathway through regulating the miR-106b-5p/LATS1 axis**. (a) Western blot of the protein levels of LATS1 and YAP were partly reversed by co-transfection with miR-106b-5p. (b) Proposed molecular mechanism of LOC107985656, which acted as a ceRNA mechanism for the miR-106b-5p/LATS1 axis and activated Hippo pathway to inhibit PTC cell proliferation. **p < 0.01; ***p < 0.001; ****p < 0.0001

Whether or not LOC107985656 activates the Hippo pathway by regulating the miR-106b-5p/LATS1 axis was further investigated. Western blot results showed that LOC107985656 overexpression markedly increased the expression of LATS1 and reduced the protein level of YAP. However, upregulation of miR-106b-5p sufficiently restored LOC107985656-induced alterations in protein levels (Figure 6(a)). Together, our results provide evidence that LOC107985656 activates the Hippo pathway by regulating the miR-106b-5p/LATS1 axis in HCC cells. We further proposed that LOC107985656 follows a ceRNA mechanism for the miR-106b-5p/LATS1 axis and activates the Hippo pathway to inhibit the proliferation of HCC cells (Figure 6(b)).

## Discussion

Increasing evidence has shown that lncRNAs are potential biomarkers or treatment targets in various human cancers, including HCC [8,25,26]. To date, the role and mechanism of LOC107985656 in cancers, particularly in HCC, are still largely unclear. Therefore, our study aims to initially investigate the potential role and molecular mechanism of LOC107985656 in HCC. We demonstrated that LOC107985656 could enhance LATS1 expression following a mechanism of lncRNA–miRNA interaction by absorbing miR-106b-5p to activate the Hippo pathway of HCC cells.

Herein, we first demonstrated that the expression of LOC107985656 was downregulated in HCC tissues and cells. Kaplan–Meier survival analysis results showed that low expression of LOC107985656 predicted a poor prognosis of patients with HCC, suggesting that LOC107985656 is a tumor suppressor gene. Further investigation demonstrated that overexpression of LOC107985656 inhibited the proliferation of HCC cells. One of the most key findings is that LOC107985656 could activate the tumor-suppressive Hippo pathway by regulating the levels of YAP and TAZ proteins. We further showed that LOC107985656 could increase the expression of LATS1 in HCC. Importantly, the mechanism by which LOC107985656 upregulates LATS1 remains unclear. Herein, we found that LOC107985656 could increase the expression of LATS1 through a ceRNA mechanism. Our findings showed that LOC107985656 increased the expression of LATS1 by absorbing miR-106b-5p in HCC cells.

The Hippo signaling pathway is involved in cell proliferation and in the progression of various diseases, including cancers [27–29]. The core Hippo signaling pathway includes LATS1/2, Ste20-like kinases 1/2 (MST1/2) and YAP and/or its paralogue TAZ [21,30]. LATS1/2, the Hippo pathway core kinase, is a tumor suppressor that reduces the oncogenic nuclear role of YAP/TAZ and TEA domain transcription factor (TEAD) [20,31]. If dephosphorylated YAP/TAZ protein is accumulated in the nucleus, the Hippo pathway is *off*, and YAP/TAZ activates gene transcription by binding to and activating TEAD and other transcription factors. If the Hippo pathway is *on*, the LATS1/2 phosphorylates YAP/TAZ on multiple sites, which may lead to cytoplasmic retention and promote the poly-ubiquitination and degradation of YAP/TAZ [32]. Herein, our results showed that p-YAP and p-TAZ were upregulated, and the expression of YAP and TAZ was downregulated by LOC107985656 overexpression, which indicates that YAP/TAZ dependent transcription was *off*. Xia et al. have reported that upregulation of lncRNA PIK3CD- AS1 inhibits the growth, invasion and metastasis of HCC cells by increasing the expression of LATS1 [33]. Yi et al. illustrated that lncRNA uc.134 inhibits the CUL4A-mediated ubiquitination of LATS1 to repress HCC progression [34]. In the current study, LOC107985656 increased the expression of LATS1 by absorbing miR-106b-5p in HCC cells. miR-106b-5p is an oncogenic miRNA in HCC [22–24]. Our results further demonstrated that LOC107985656 activated the Hippo pathway through the miR-106b-5p/LATS1 axis in HCC cells.

## Conclusion

In summary, our study first reported that LOC107985656 was downregulated in HCC tissues and cells. Further investigations have shown that LOC107985656 could inhibit HCC proliferation by regulating the miR-106b-5p/LATS1 axis and activating the Hippo pathway. Our results might provide insights into the development of HCC and propose lncRNA-directed diagnosis and therapy for HCC.

## Supplementary Material

Supplemental Material

## Data Availability

The data used to support the findings of this study are available from the corresponding author upon request.

## Article Highlights

1) LOC107985656 was downregulated in HCC cells and tissues.

2) LOC107985656 repressed cell proliferation and metastasis of HCC cells.

3) LOC107985656 activated the Hippo pathway through regulating the miR-106b-5p/LATS1 axis.
