# Supplementary material for: Long non-coding RNA LOC107985656 represses the proliferation of hepatocellular carcinoma cells through activation of the tumor-suppressive Hippo pathway
Source: Bioengineered. 2021 Oct 8;12(1):7964–74. doi: 10.1080/21655979.2021.1984005 (PMC8806957; doi:10.1080/21655979.2021.1984005)
Supplement: Supplemental Material [file KBIE_A_1984005_SM7118.doc]

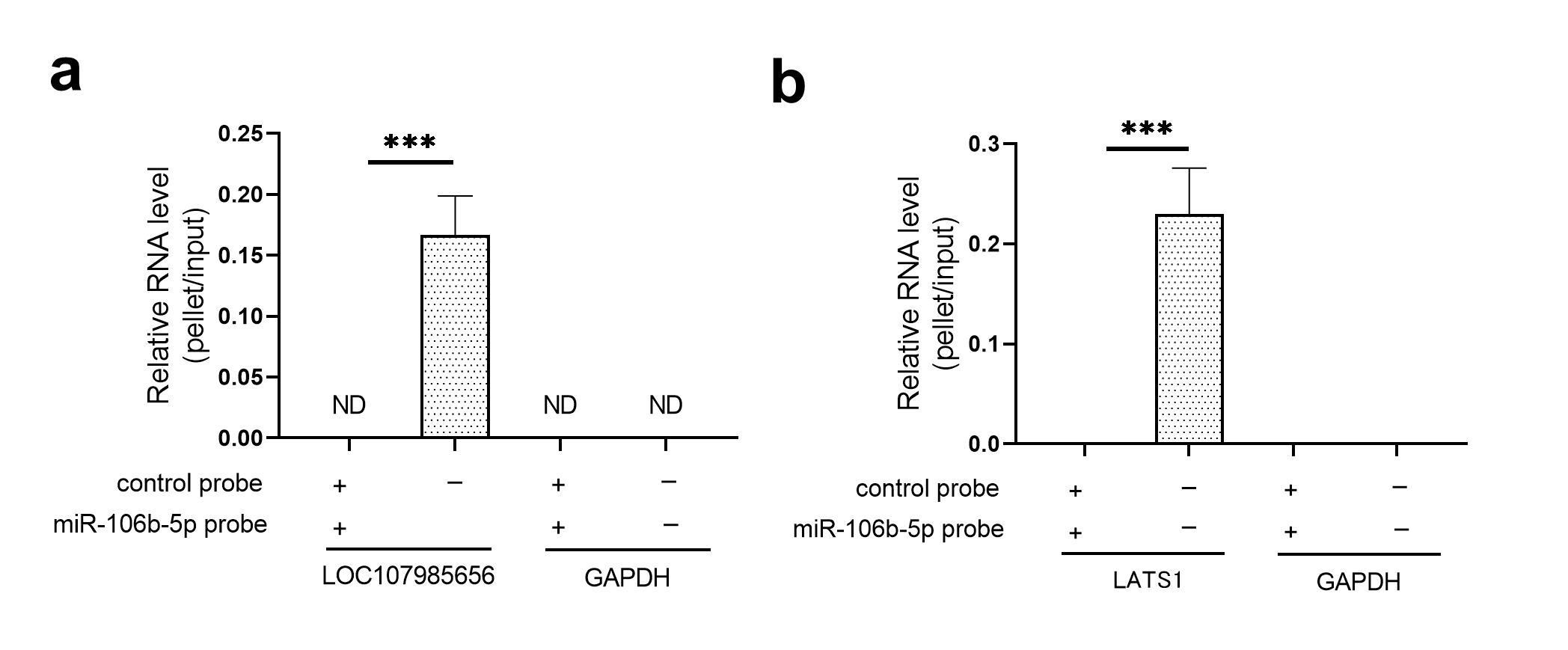


Figure S1: RNA pull-down in huh7 cells transfected with biotin-labeled miR-106b-5p mimics, followed by RT-qPCR analysis. (a) RNA pull-down exhibited an interaction between miR-106b-5p and LOC107985656. (b) RNA pull-down exhibited an interaction between miR-106b-5p and LATS1. ****p*<0.001.
